# Reovirus inhibits interferon production by sequestering IRF3 into viral factories

**DOI:** 10.1038/s41598-017-11469-6

**Published:** 2017-09-07

**Authors:** Megan L. Stanifer, Christian Kischnick, Anja Rippert, Dorothee Albrecht, Steeve Boulant

**Affiliations:** 1 Department of Infectious Diseases, Virology, Heidelberg University, Heidelberg, Germany; 2 Schaller research group at CellNetworks and German Cancer Research Center (DKFZ), Research Group “Cellular polarity and viral infection” (F140), Heidelberg, Germany

## Abstract

Upon viral infection, an arms-race between the cellular intrinsic innate immune system and viral replication is established. To win this race, viruses have established multiple strategies to inhibit the cellular response. Mammalian reovirus (MRV) constitutes a great model to study pathogenesis and life cycle of dsRNA viruses. It replicates in the cytosol of infected cells by forming viral induced-replication compartments, or viral factories. Little is known about the strategy used by MRV to evade the cellular intrinsic immune system. In this study, we unraveled that MRV induces a replication-dependent global reduction in interferon-mediated antiviral immune response. We determined that although MRV leads to the activation and phosphorylation of interferon regulatory factor 3 (IRF3), the nuclear translocation of IRF3 was impaired in infected cells. Additionally, we showed that MRV does not degrade IRF3 but sequesters it in cytoplasmic viral factories. We demonstrate that the viral factory matrix protein μNS is solely responsible for the sequestration of IRF3. This finding highlights novel mechanisms used by MRV to interfere with the intrinsic immune system and places the viral factories as not only a replication compartment but as an active strategy participating in immune evasion.

## Introduction

From the onset of viral infection, a race between cellular strategies to inhibit viral replication/spread and viral strategies to directly interfere with these cellular mechanisms is initiated. The outcome of viral infection: successful replication with spread *vs*. viral eradication is the direct consequence of these two conflicting strategies. The cellular host has developed numerous mechanisms to counteract pathogens. The cellular proteins that detect the presence of foreign constituents are referred as pattern-recognition receptors (PRRs). The most important viral constituent or pathogen-associated molecular pattern (PAMPs) that is sensed during infection is the viral nucleic acid. The cytosolic RIG-like receptor (RLR) proteins RIG-I and MDA5 and the endosomal Toll-like receptor 3 (TLR3), sense viral RNA. Upon sensing of viral RNA, RLR proteins form together with the MAVS protein located on the surface of mitochondria and peroxisomes^1^ a signaling platform that induces the activation of a complex signaling cascade while TLR3 forms a complex with TRIF, which establishes a similar signaling cascade^2, 3^. This signaling ultimately leads to the activation of both the NF-κB and the interferon regulatory factor 3 (IRF3) pathways which upon activation translocate in the nucleus where they act as transcription factors to induce the production of interferons (IFNs), a subset of interferon stimulated genes (ISGs), and pro-inflammatory cytokines. These cytokines, in turn, act in a paracrine and autocrine manner to combat viral infection and to provide naïve cells an antiviral state that will limit viral infection spread. These mechanisms constitute the intrinsic innate immune system present in all cells^4^.

In response to these defense mechanisms, viruses have evolved and established various strategies to inhibit these signaling cascades. Many viruses encode specific viral proteins that either block activation, induce degradation, or sequester key cellular proteins of theses signaling pathways. Other viruses interfere with restriction factors to create a better cellular environment that is more permissive for their replication^5^. An alternative strategy developed by viruses is to keep under the radar of the cellular intrinsic innate immune system. For the cytosolic RNA viruses, many form viral-induced replication compartments or viral factories (VFs)^6^. These VFs act as a protective microenvironment in which viruses replicate their genomes and assemble progeny viral particles^7^. It has been suggested that these VFs, by retaining viral constituents (viral nucleic acids and proteins) in a confined environment, act as a protective barrier to avoid sensing by the intrinsic innate immune system.

Mammalian reoviruses (MRV) are non-enveloped viruses with a segmented double stranded RNA (dsRNA) genome from the family *Reoviridae*, which contain the clinically important human and animal stock pathogens rotavirus and bluetongue virus, respectively^8, 9^. MRV represents a useful model to study dsRNA virus replication and pathogenesis. MRV virions bind to cells using sialic acid, integrins, and JAM-A^10–13^. They are internalized by a combination of micropinocytosis and clathrin-mediated endocytosis and are transported to the endosomal pathway^14, 15^. In the late endosomes, virions encounter the serine proteases cathepsins, which convert virions into intermediate subviral particles (ISVPs)^16–18^. ISVPs further convert within the endosomes to mediate the release of the transcriptionally active core particle into the cytosol of the infected cell^19, 20^. MRV is a respiratory and enteric virus and its first barrier to infection are epithelial cells. During fecal-oral transmission, within the gastro-intestinal tract, MRV virions are converted by serine proteases into ISVPs prior to infection^21^. As such ISVPs initiate the first round of MRV infection in the gastro-intestinal tract. The subsequent rounds of infections, as the virus spreads within the host, are mediated by virions. We have previously demonstrated that initiating infection by ISVPs provides MRV multiple advantages over their virion counterparts. ISVPs are more infectious and lead to the production of TGF-β, which acts as a protective factor and allows for cell survival. On the contrary, infection by virions does not cause TGF-β secretion and cells rapidly die after infection^22^.

As dsRNA viruses, MRV virions are sensed by both TLR3 and the RLR pathway. On the contrary, ISVPs are only sensed by the RLR pathway^22, 23^. Like many other viruses, *Reoviridae* have developed mechanisms to counteract the intrinsic innate immune response. Rotaviruses are the best characterized among the family. They have developed a strategy to degrade many members of the IRF family (including IRF3) by the proteasome, and are as such interfering globally with the IFN-mediated antiviral response^24, 25^. In the case of MRV, much less is known. The µ2 protein has been described to interfere with the transcription factors IRF9 and IRF7, but the mechanisms underlying the repression are not clear^26–28^. Similarly, σ3 protein appears to modulate the type I IFN response by inhibiting the dsRNA-mediated activation of PKR^29^.

Here, we address whether MRV has developed mechanisms leading to a global inhibition of IFN signaling, like that observed for rotavirus. We show that MRV induces a replication-dependent mechanism that drives the inhibition of the cellular antiviral innate immune response. This mechanism, characterized by the inhibition of IFN production, is not only restricted to the reovirus-induced immune response but also inhibits the immune response generated upon infection by non-related viruses or stimulation with unrelated PAMPs. We demonstrate that MRV does not interfere with IRF3 activation nor does it induce IRF3 degradation but instead sequesters it into viral factories. We identify µNS, the viral protein responsible for the formation of MRV viral factories, as the sole viral protein able to sequester IRF3. This prevents IRF3 translocation into the nucleus thereby blocking the production of IFNs. These results demonstrate a novel mechanism developed by MRV to win the race against the cellular intrinsic innate immune system. Importantly, our results clearly demonstrate, for the first time, a VF-driven mechanism to escape the cells intrinsic innate immune system.

## Results

### ISVPs induce less antiviral immune response compared to virions

**Figure 1:**
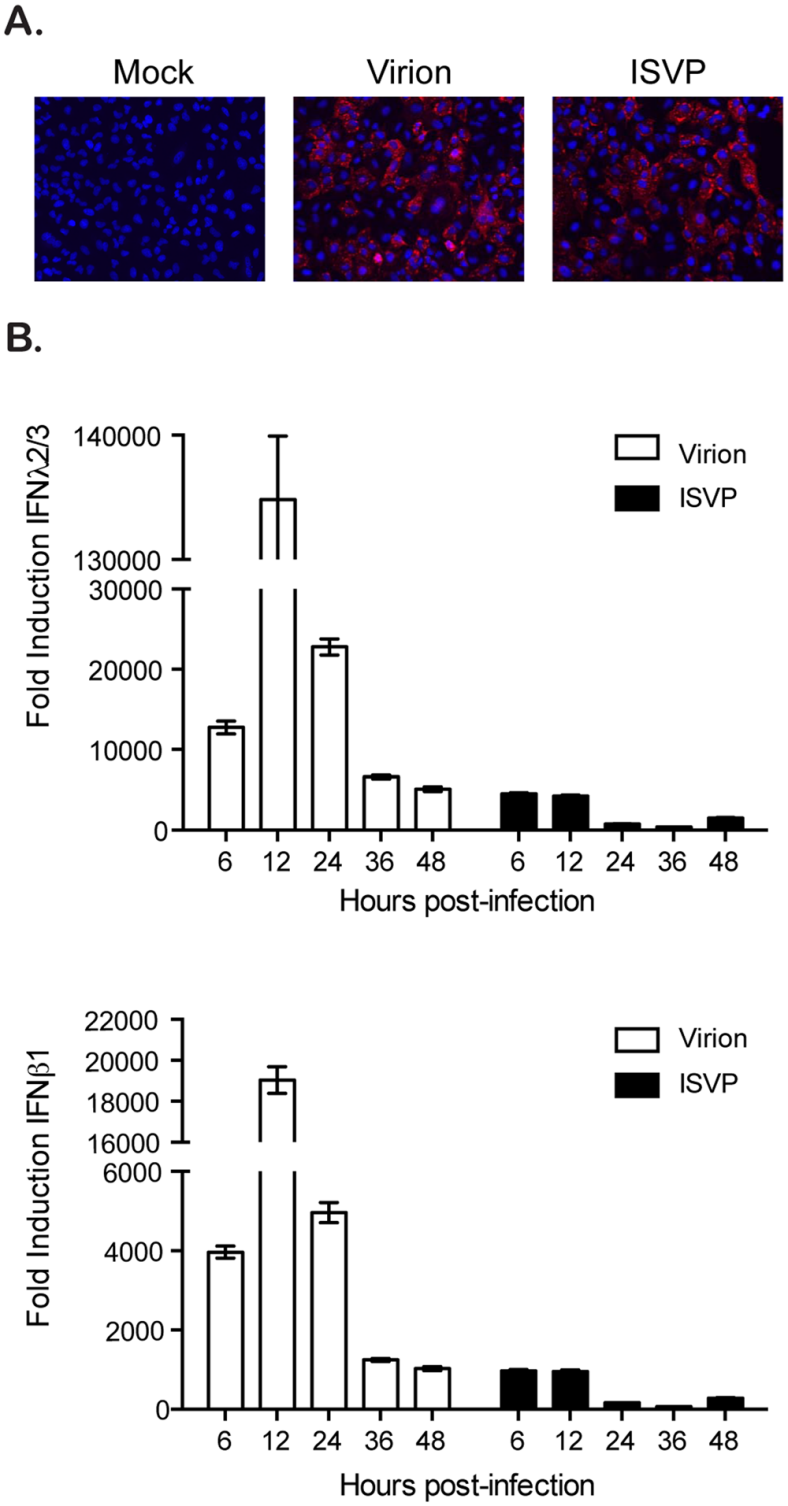
ISVP infection induces less innate immune response compared to virion infection. A549 cells were infected with reovirus MRV virions or ISVPs at MOI = 1. (**A**) Virus infection was monitored 12 hpi by fluorescence microscopy following immunostaining of the non-structural viral protein μNS (red). Cell nuclei were stained using DAPI (blue). Experiment was performed in triplicate; representative images are shown. (**B**) At indicated times post-infection of A549 cells by virions or ISVPs, the transcriptional upregulation of IFNβ1 and IFNλ2/3 was monitored by qRT-PCR. Results are normalized to mock infected cells for each time point. Experiment was performed in triplicate; average and standard deviation are shown.

Our previous work determined that MRV ISVPs induce a lower immune response during infection of cells compared to their virion counterparts^22^. We proposed that this lower immune response and reduced production of protective cytokines give ISVPs an infectious advantage compared to virions. To better characterize the mechanisms by which ISVPs avoid recognition by the innate immune system or by which they inhibit the innate immune system, the lung epithelial cells A549 were infected with either MRV virions or ISVPs using a multiplicity of infection (MOI) of 1. 12 hours post-infection (hpi), A549 cells were fixed and infected cells were identified by immunostaining for the non-structural protein μNS. (Fig. 1A). Interestingly, monitoring the antiviral immune response by measuring the transcriptional upregulation of IFNβ1 and IFNλ2/3 during the course of MRV infection confirmed that ISVPs induce less antiviral immune response compared to virions (Fig. 1B). This observation suggests that ISVPs have developed strategies to bypass or to inhibit the intrinsic innate immune system of infected cells.

### Replication is required for ISVP-mediated inhibition of antiviral response

**Figure 2:**
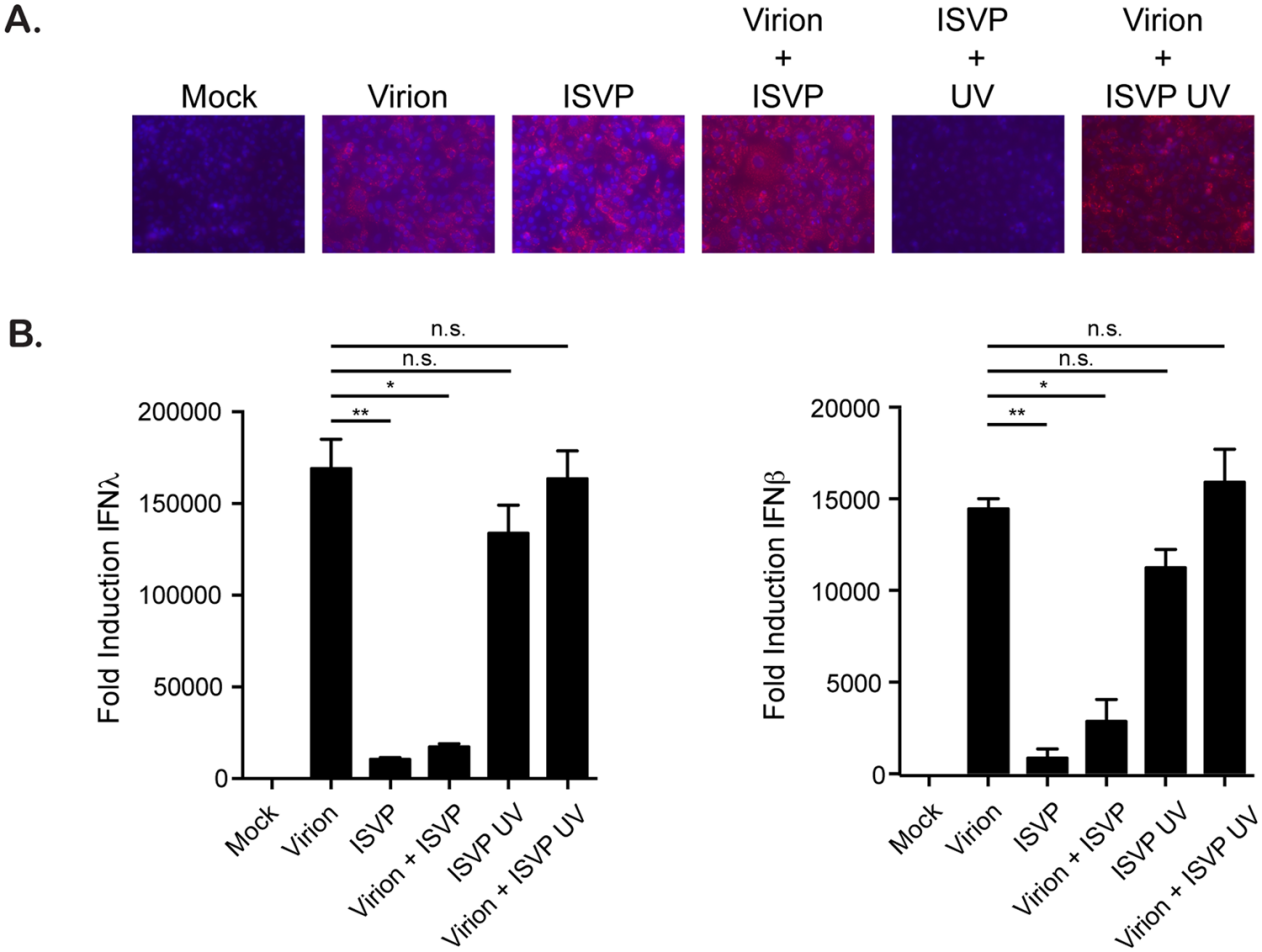
Replication is required for ISVPs to interfere with immune response. A549 cells were infected with MRV virions and/or ISVPs at an MOI of 1. ISVPs were used mock treated or UV inactivated. In co-infection experiments, virions and ISVPs were each used at an MOI of 1. (**A**) Virus infection was monitored 16 hpi by fluorescence microscopy following immunostaining of the non-structural viral protein μNS (red). Cell nuclei were stained using DAPI (blue). Experiment was performed in triplicate; representative images are shown. (**B**) The transcriptional upregulation of IFNβ1 and IFNλ2/3 was monitored by qRT-PCR at 16 hpi. Results are normalized to mock infected cells. Experiment was performed in triplicate; average and standard deviation are shown. *P < 0.05 **P < 0.01 (unpaired t-test).

To determine whether the lower antiviral immune response induced by ISVPs was due to either lower detection efficiency of ISVP infection by the immune system or to an ISVP-specific downregulation of the innate immune response, we performed co-infection experiments of cells with both virions and ISVPs. 16 hpi, infection was confirmed by indirect immunofluorescence for the non-structural protein μNS (Fig. 2A) and the extent of the immune response was evaluated by monitoring the transcriptional upregulation of both IFNβ1 and IFNλ2/3 (Fig. 2B). As expected ISVPs induced drastically less production of both IFNs compared to virions (Fig. 2B). Interestingly, although cells co-infected with both virions and ISVPs display a greater number of infected cells compared to virions or ISVPs alone (Fig. 2A), they show a significant reduction in IFN production compared to virions alone (Fig. 2B). This observation suggests that ISVPs directly interfere with IFN production. To determine whether this inhibitory mechanism requires replication, ISVPs were inactivated using UV and were then evaluated for their ability to restrict the virion-induced immune response. Failure to detect the production of the non-structural protein μNS confirmed that the UV-inactivated ISVPs were not infectious (Fig. 2A). Interestingly, UV-inactivated ISVPs induced a greater immune response compared to their replication competent counterparts (Fig. 2B). Most importantly, we found that they were unable to interfere with the virion-induced immune response in co-infection experiments (Fig. 2B). These results indicate that replication is mandatory for ISVPs to inhibit the production of IFNs.

### ISVP co-infection interferes with the immune response generated by cells stimulated by various pathogen-associated molecular patterns

**Figure 3:**
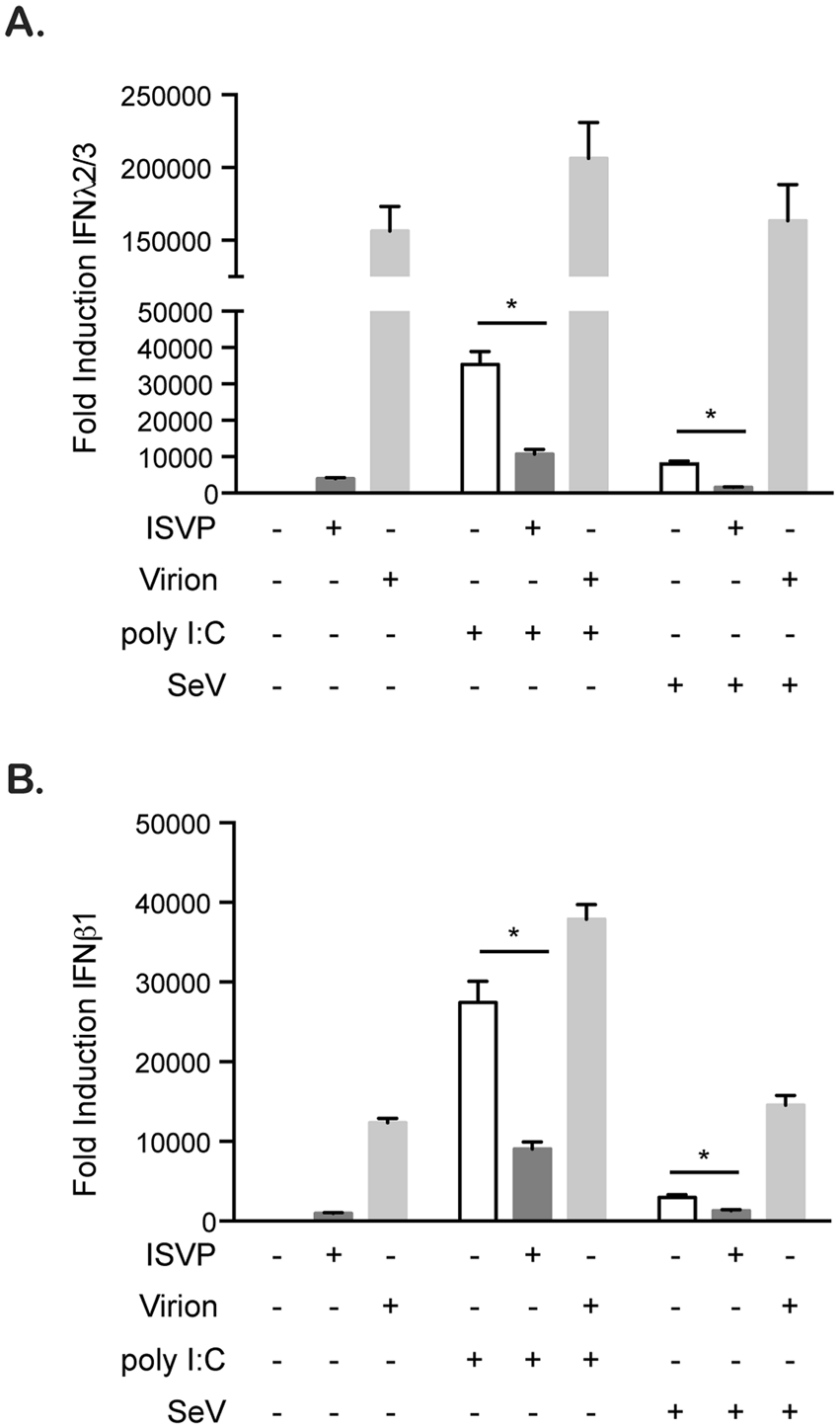
ISVPs interfere with SeV- and poly I:C-induced immune response. A549 cells were mock treated, treated with poly I:C (1 µg/mL), or infected with Sendai Virus (SeV) (MOI = 3) in the presence (+ISVP) or absence (−ISVP) of ISVP at MOI = 1. The transcriptional upregulation of IFNβ1 and IFNλ2/3 was monitored by qRT-PCR at 16 hpi. Results are normalized to mock treated and mock infected cells. Experiment was performed in triplicate; average and standard deviation are shown. *P < 0.05 (unpaired t-test).

To determine whether the ISVP-induced inhibition of innate immune response was restricted to reovirus infection or whether it could also interfere with the immune response induced by other pathogen-associated molecular patterns (PAMPs) or by other non-related viruses, we evaluated the effect of ISVPs co-infection together with transfection of poly I:C or with Sendai virus (SeV) infection. A combination of both high and low molecular weight poly I:C was transfected into A549 cells to stimulate the RIG-I and MDA5 pathway. As expected, poly I:C stimulated the induction of both IFNβ1 and IFNλ2/3 transcripts (Fig. 3A and B). Interestingly, when poly I:C transfected cells were co-infected with ISVPs, the induction of both IFNβ1 and IFNλ2/3 was significantly reduced (Fig. 3A and B). SeV is a negative sense single-stranded RNA virus from the *Paramyxoviridae* family and is a known stimulator of RIG-I. Infection of A549 cells with SeV resulted in the transcriptional upregulation of both IFNβ1 and IFNλ2/3 compared to mock infected cells (Fig. 3A and B). Similarly to poly I:C treatment, co-infection of SeV-infected cells with ISVPs caused a significant reduction of both IFNβ1 and IFNλ2/3 production (Fig. 3A and B). Interestingly, co-infection of virion with poly I:C or SeV showed additive effect in antiviral immune induction suggesting that this down regulation is specific to ISVPs (Fig. 3A and B). Together these results show that ISVPs can globally interfere with the immune response generated by cells stimulated by various PAMPs.

### ISVPs and virions activate the cell intrinsic immune system but interfere with interferon regulatory factor 3 nuclear translocation

**Figure 4:**
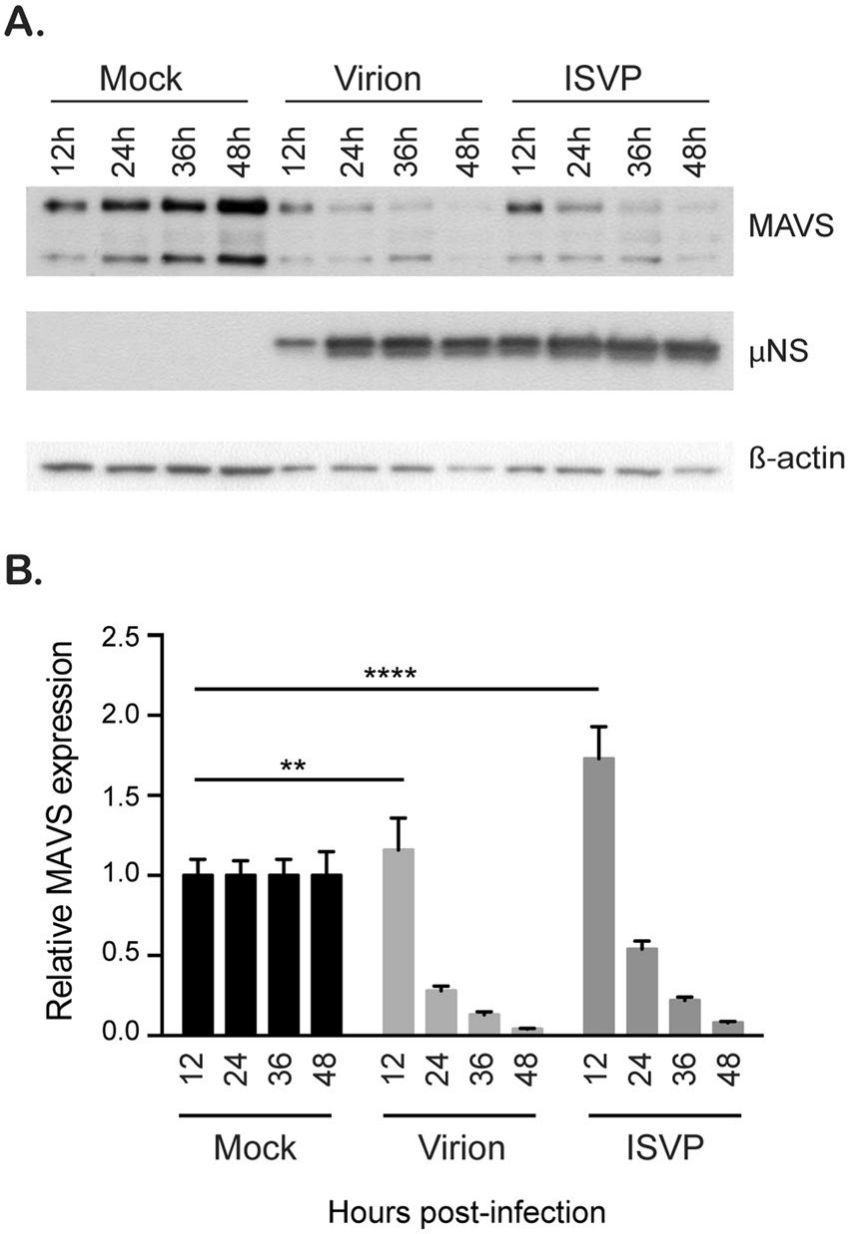
Reovirus MRV virions and ISVPs cause MAVS degradation. A549 cells were mock infected or infected with virions or ISVPs at MOI = 3. In 12-hour intervals, mock and infected cells were collected. Infection and MAVS degradation was followed by Western blot by monitoring the production of the non-structural viral protein μNS and the total amount of MAVS in the cell lysate, respectively. β-actin is used as a loading control. (**A**) Representative Western blot from an experiment performed in triplicate. Blots were cropped and full blots can be found in supplementary materials. (**B**) Relative MAVS expression was quantitated by densitometry of the Western blot in (**A**) Data are normalized to mock infected for each time points. Experiment was performed in triplicate; average and standard deviation are shown. **P < 0.01, ****P < 0.0001 (unpaired t-test).

As virions and ISVPs are both sensed by the RLR pathway whereas virions can also be sensed by TLR3^22^, we next wanted to address whether the observed ISVP-mediated interference with the immune response was due to an inhibition of RLR signaling. A hallmark of prolonged activation of the RLR pathway is the degradation of the mitochondrial antiviral signaling protein (MAVS)^30^. To determine if ISVPs interfere with MAVS activation and/or degradation, A549 cells were infected with MRV virions or ISVPs. At different times post-infection, protein lysates were harvested and analyzed for the presence of the MAVS protein and MRV infection. In ISVP infected cells, we noticed an earlier accumulation of μNS compared to virion-infected cells. This observation corroborates with the fact that ISVP infection is faster than virion-mediated infection (Figs 4A and S1 and ref. 14). At 12 hpi, for both virion and ISVP infected cells, a slight increase in the amount of MAVS was observed particularly during ISVP infection (Fig. 4A and B). This accumulation of MAVS is the result of MAVS being synthesized in response to viral infection. The MAVS protein is known to be an interferon stimulated gene (ISG) whose expression is promoted by IFN mediated signaling. Interestingly, both virions and ISVPs led to similar kinetics of MAVS degradation from 24 to 48 hpi (Fig. 4A and B). These results suggest that both virion and ISVP infection equally lead to MAVS activation. As such, this finding supports a model where ISVPs do not interfere with immune response by subverting their detection by the RIG-I/MDA5/MAVS signaling pathway.

**Figure 5:**
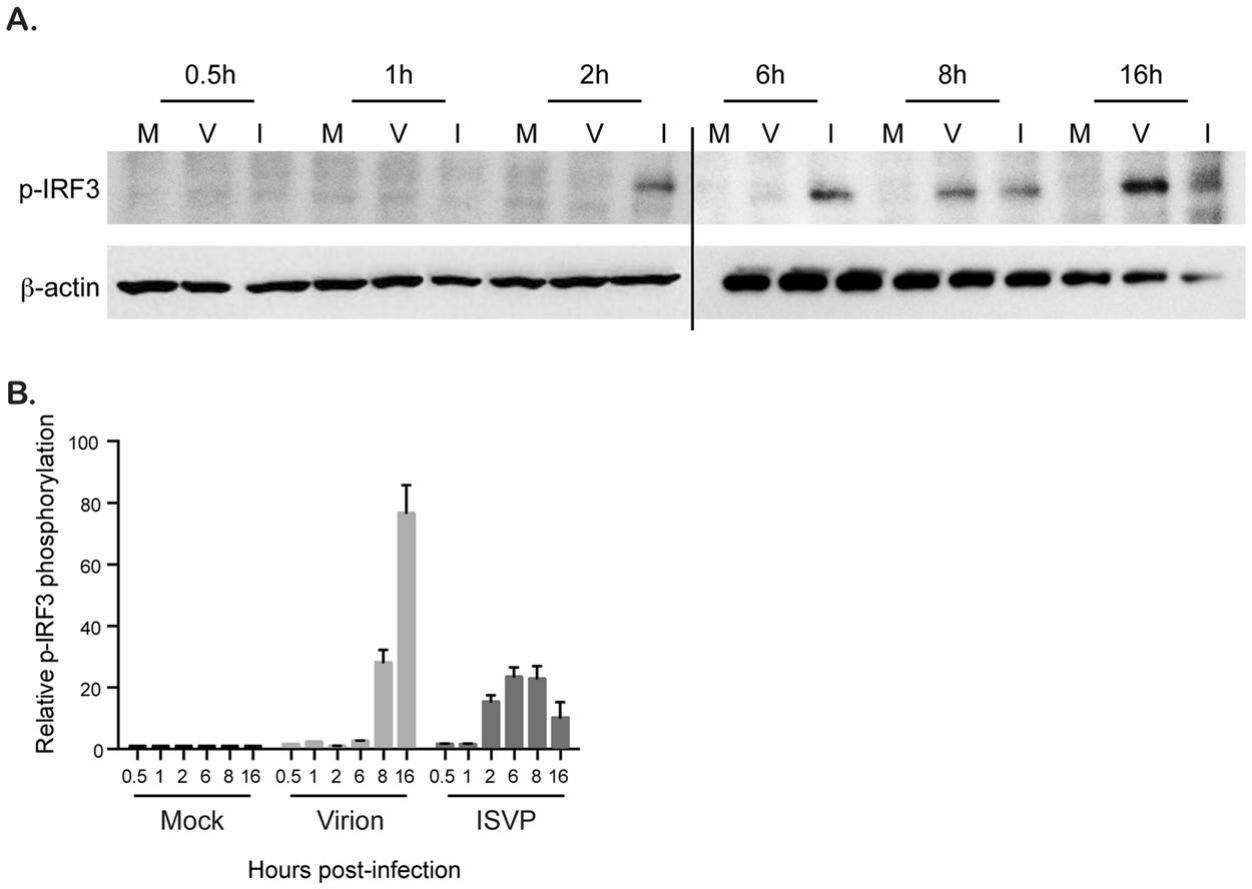
Reovirus virion and ISVP infection induce phosphorylation of IRF3. (**A**) A549 cells were mock infected or infected with virions or ISVPs at MOI = 3. Protein lysates were harvested at indicated times and the phosphorylation status of IRF3 was monitored by Western blot using an anti-phospho-IRF3. β-actin is used as a loading control. Experiment was performed in triplicate; Representative Western blot is shown. Two blots were run to accommodate all samples, black line indicates break in blots. Blots were cropped and full scans can be found in supplementary material. (**B**) Relative phospho-IRF3 expression was quantitated by densitometry of the Western blot in (**A**). Data are normalized to mock infected for each time points. Experiment was performed in triplicate; average and standard deviation are shown.

Following sensing of viral infection by RIG-I/MDA5 and activation of MAVS, TBK1 is activated which leads to the phosphorylation of the interferon regulatory factor 3 (IRF3). Once phosphorylated, IRF3 translocates into the nucleus and acts as transcription factor to induce the transcription of IFNs and some ISGs^31^. IRF3 has been previously shown to be activated and translocated into the nucleus in virion infected samples^32^. To confirm this previous report and to determine if ISVPs also activate IRF3, A549 cells were infected with virions and ISVPs and IRF3 phosphorylation was monitored over time. Interestingly, both virion and ISVP infection induced the phosphorylation of IRF3 (Fig. 5A). ISVPs caused IRF3 phosphorylation by 2 hpi and this phosphorylation was maintained until 8 hpi (Fig. 5A and B). Virion-induced IRF3 phosphorylation was delayed and detected around 8 hpi (Fig. 5A and B). This delayed IRF3 activation compared to ISVP infection was expected as virions require a longer time to be delivered to the cytosol due to their need for endosomal cathepsin processing (Figure S1 and ref. 14). These results show that both virion and ISVP infection induce the activation of IRF3.

**Figure 6:**
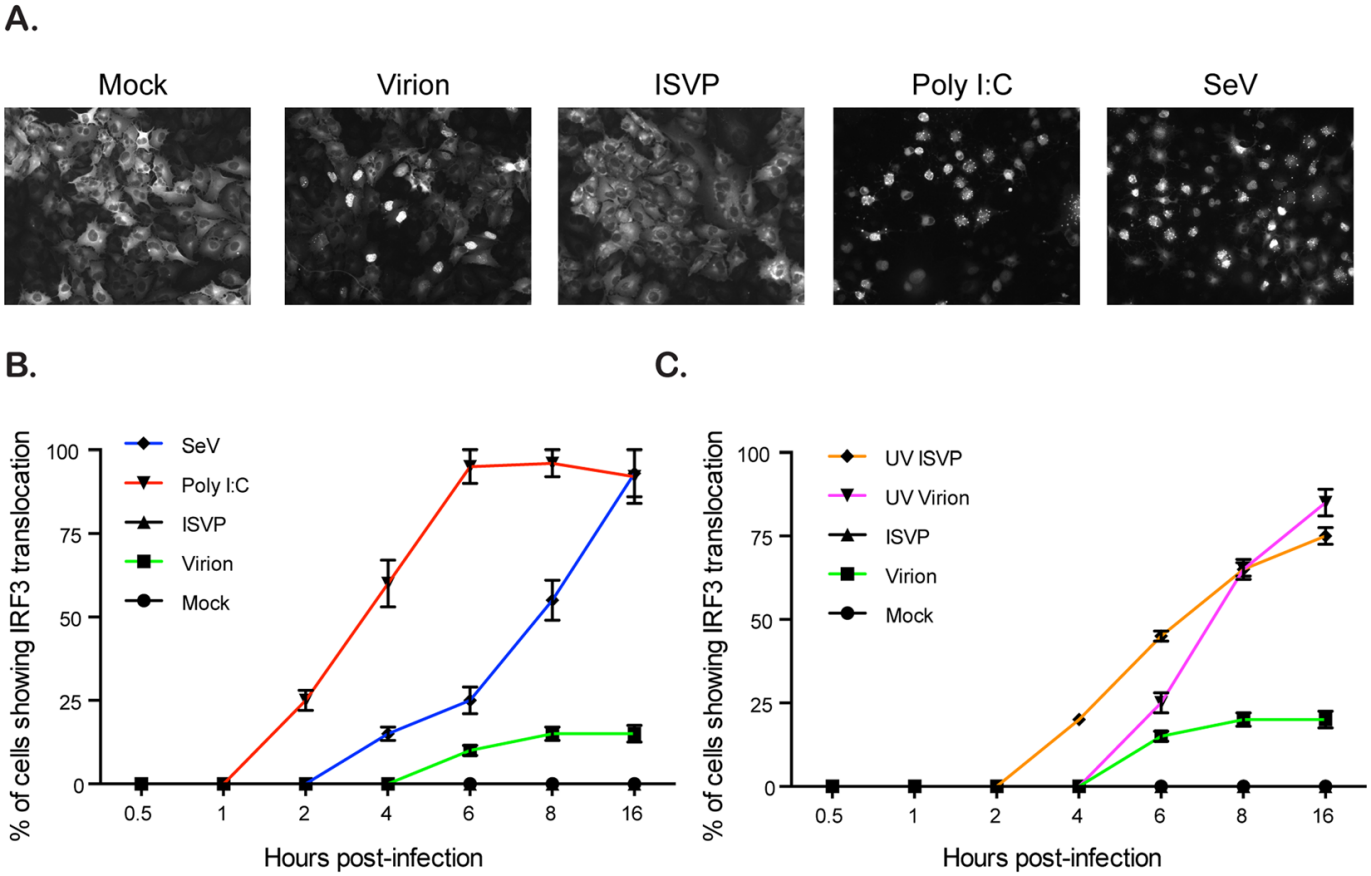
IRF3 nuclear translocation is inhibited in ISVP infected cells. A549 cells expressing IRF3-GFP were mock infected, infected with virions or ISVPs (active or UV-inactivated) at MOI = 3, or with SeV at MOI = 3 or were transfected with 1ug/mL of poly I:C. (**A**) IRF3-GFP nuclear translocation was monitored 16 hpi/transfection by fluorescence microscopy. Experiment was performed in triplicate, representative images are shown. (**B** and **C**) IRF3-GFP nuclear translocation was monitored at indicated time points post infection/transfection by fluorescence microscopy. The relative number of cells displaying IRF3-GFP in the nucleus is normalized to the total number of IRF3-GFP expressing cells. The numbers of cells displaying nuclear translocation were counted in six fields of view per sample/time point/replicate (with around 200 cells/field of view). The total number of cells showing nuclear translocation (sum of all time points) in mock = 0, ISVP = 0, virion = 1443, SeV = 6837, poly I:C = 12,986, UV-virion = 7377, and UV-ISVP = 6298. Experiment was performed in triplicate; average and standard deviation are shown.

As IRF3 only stimulates the production of IFNs following its translocation into the nucleus, we evaluated whether virion and ISVP infection lead to similar kinetic of IRF3 nuclear translocation. A549 cells expressing IRF3-GFP were either mock, virion, or ISVP infected using an MOI of 3. Poly I:C and SeV, well characterized inducers of RLR mediated signaling, were used as positive control. Mock infected cells showed IRF3-GFP evenly distributed in the cytosol (Fig. 6A). As expected, A549 cells stimulated with poly I:C or infected with SeV showed complete IRF3 nuclear translocation by 6 h post-treatment and 16 hpi, respectively (Fig. 6A and B). By 16 hpi, although all cells were infected by virions, only around 20% of virion-infected cells showed nuclear localization of IRF3 (Fig. 6A and B). Interestingly, ISVP infected cells showed no IRF3-GFP nuclear translocation (Fig. 6A and B). As we previously demonstrated that ISVPs required active replication to inhibit the antiviral immune induction (Fig. 2), we evaluated whether UV inactivated ISVPs were able to block the nuclear import of IRF3. Interestingly, UV inactivated ISVPs showed nuclear translocation of IRF3 (Fig. 6C). Importantly, infection of cells with UV inactivated virions resulted in a near complete nuclear translocation of IRF (Fig. 6C). All together these results show that both virions and ISVPs are detected by the innate immune system and induce phosphorylation of IRF3. However, virions appear to have developed a mechanism to partially block IRF3 transport into the nucleus while ISVP can fully inhibit IRF3 nuclear translocation.

### IRF3 is sequestered in viral factories

**Figure 7:**
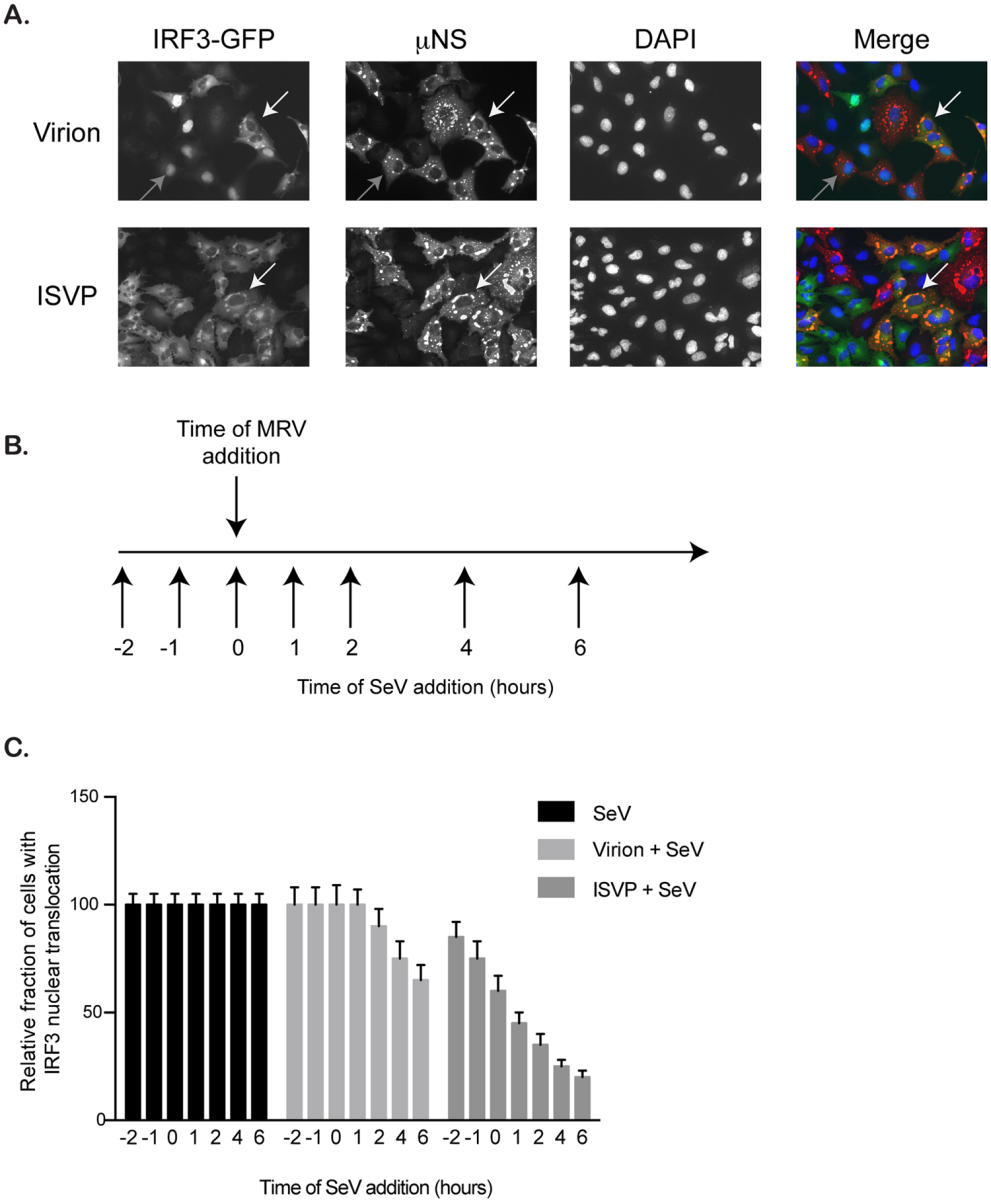
IRF3 localizes to reovirus viral factories. (**A**) A549 cells expressing IRF3-GFP were infected with virions or ISVPs at MOI = 1. 16 hpi cells were fixed and immunostained for the non-structural protein μNS (red). Gray arrows indicate nuclear translocated IRF3-GFP, white arrows indicate IRF3-GFP associated with viral factories. Cell nuclei were stained using DAPI (blue). Experiment was performed in triplicate. Representative images are shown. (**B**) Schematic showing the experimental approach: A549 cells expressing IRF3-GFP were infected with virions or ISVPs at MOI = 3. At indicated times pre- or post-reovirus infection (t = 0 represents reovirus infection time), Sendai virus (SeV) was added to the reovirus infected cells at MOI = 3. (**C**) Same as B except at 16 h post-MRV infection, IRF3-GFP nuclear translocation was monitored by fluorescence microscopy. The relative number of cells displaying IRF3-GFP in the nucleus is normalized to the total number of IRF3-GFP expressing cells. The numbers of cells displaying nuclear translocation were counted in six fields of view per sample/time point/replicate (with around 150 cells/field of view). The total number of cells showing nuclear translocation (sum of all time points) in SeV = 12611, SeV+virion = 11358, SeV+ISVP = 6207. Experiment was performed in triplicate; average and standard deviation are shown.

MRV replication and assembly takes place in virally induced cytosolic compartments referred to as viral factories (VFs). We have previously shown that these viral factories can sequester many important cellular factors^33^. To determine if IRF3 can also be a target for sequestration in VFs, A549 cells expressing IRF3-GFP were infected with MRV virions or ISVPs. 16 hpi, cells were fixed and stained for the non-structural protein μNS. In virion-infected cells, IRF3 can be found either in the nucleus (Fig. 7A, gray arrows) or trapped in VFs (Fig. 7A, white arrows). Importantly, in ISVP infected cells, IRF3 was never found in the nucleus and was always associated with μNS within the VFs (Fig. 7A, white arrows). To confirm that sequestration of IRF3-GFP into MRV VFs was not due to the GFP moiety, we used A549 expressing GFP as control. Infection of GFP-expressing A549 cells by either virions or ISVPs did not lead to the sequestration of GFP into VFs (Figure S2A). Additionally, to ensure that sequestration of IRF3 into VFs was not the results of its overexpression in A549 cells, we performed immunofluorescence staining against endogenous IRF3. While IRF3 normally localized in the cytosol of non-infected cells, we could clearly detect IRF3 into MRV VFs upon either virion or ISVP infection (Figure S2B). Additionally, as μNS has not previously been shown to be a genetic determinant of immune response, we confirmed that the capacity of μNS to sequester IRF3 was strain independent. A549 cells expressing IRF3-GFP display similar sequestration of the transcription factor into VFs upon infection with the MRV type 1 Lang (T1L) strain (Figure S3).

ISVPs can downregulate both the poly I:C and SeV-induced innate immune response (Fig. 3). To determine whether this inhibition was the result of IRF3 sequestration in MRV VFs, co-infection experiments between ISVPs and SeV were performed. SeV was added to ISVP infected cells in 1 hour intervals starting 2 hours prior to ISVP infection (Fig. 7B). As expected, SeV infection resulted in IRF3 nuclear translocation in all infected cells. Interestingly, co-infection with ISVPs could block the nuclear accumulation of IRF3 in a time dependent manner. The longer the cells were infected with ISVP prior SeV infection, the more IRF3 nuclear translocation was inhibited (Fig. 7C). Similar results were obtained when cells were co-infected with virions and SeV. However, the virion-mediated inhibition of IRF3 nuclear translocation was less pronounced and delayed compared to ISVP co-infection (Fig. 7C). This was in full agreement with the observed inhibition of IRF3 nuclear translocation during virion and ISVP infection (Fig. 6). These results suggest that MRV interfere with the innate immune response by sequestering IRF3 in VFs.

**Figure 8:**
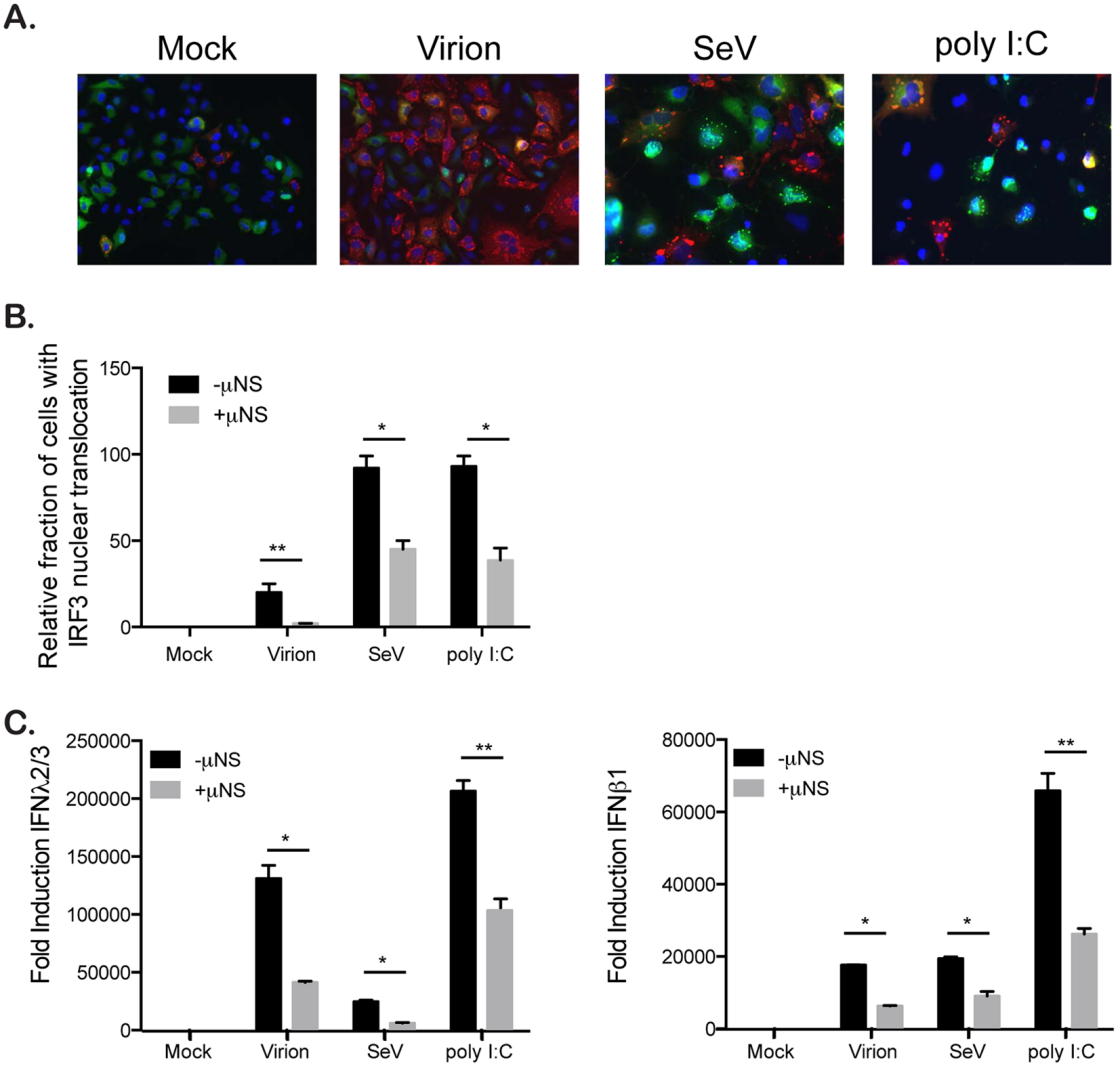
The viral factory protein μNS sequesters IRF3 reducing antiviral innate immune induction. A549 expressing IRF3-GFP cells were transduced with a BacMam expressing the viral factory protein μNS or GFP. 24 hours post-transduction, cells were mock infected or infected with virions at MOI = 3, SeV at MOI = 3 or transfected with poly I:C. 16 hours post-infection/transfection, cells were harvested and analyzed for IRF3-GFP nuclear translocation and inhibition of IFN production. (**A** and **B**) IRF3-GFP nuclear translocation was monitored at indicated time points post infection/transfection by fluorescence microscopy. The viral factory protein μNS was immunostained using an anti-μNS antibody (red). The relative number of cells displaying IRF3-GFP in the nucleus is normalized to the total number of IRF3-GFP expressing cells. The numbers of cells displaying nuclear translocation were counted in six fields of view per sample/replicate. The total number of cells showing nuclear translocation in virion(−)μNS = 144, virion(+)μNS = 16, SeV(−)μNS = 645, SeV(+)μNS = 321, poly I:C(−)μNS = 672,poly I:C(+)μNS = 385. Experiment was performed in triplicate; average and standard deviation are shown. *P < 0.05, **P < 0.01 (unpaired t-test) (**C**) The transcriptional upregulation of IFNβ1 and IFNλ2/3 was monitored by qRT-PCR at 24 hpi. Results are normalized to mock infected cells. Experiment was performed in triplicate; average and standard deviation are shown. *P < 0.05, **P < 0.01 (unpaired t-test).

**Figure 9:**
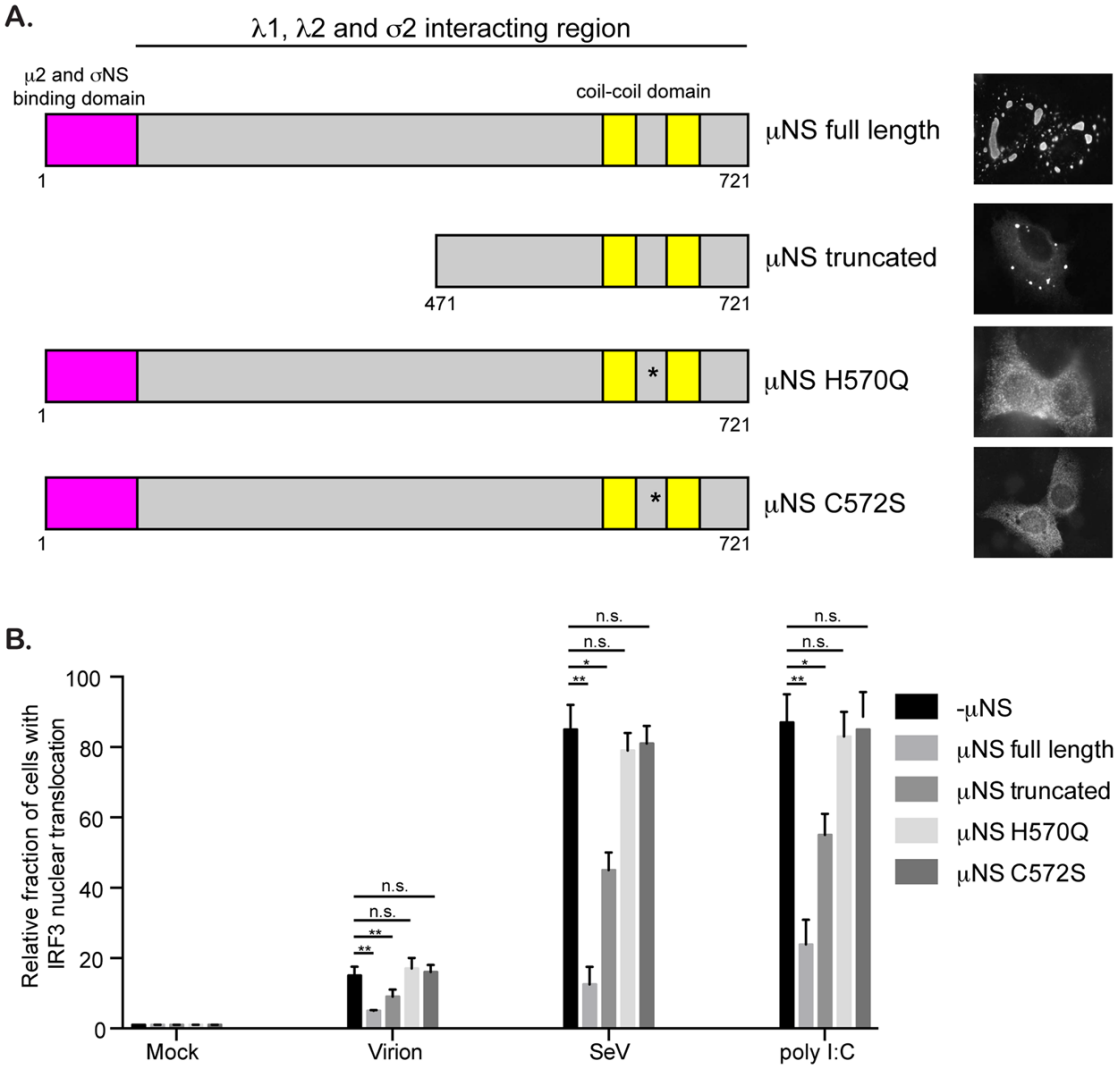
IRF3 sequestration is dependent on μNS’s capacity to form viral factories. (**A**) Schematic showing the various μNS constructs and their cytoplasmic distribution. (**B**). A549 expressing IRF3-GFP cells were transduced with lentiviruses expressing μNS constructs as described in A. -μNS cells are cells which were transduced with a RFP control protein. 72 hours post-transduction, cells were infected with MRV virions (MOI = 3), SeV (MOI = 3) or transfected with poly:IC. 16 hours post-infection/transfection IRF3-GFP nuclear translocation was monitored by fluorescence microscopy. The viral factory protein μNS was immunostained using an anti-μNS antibody. The relative number of cells displaying IRF3-GFP in the nucleus is normalized to the total number of μNS expressing cells. The numbers of cells displaying nuclear translocation were counted in 10 fields of view per sample/replicate (with around 150 cells/field of view). The total number of cells showing nuclear translocation in virion(−)μNS = 93, virion(+)μNS full length = 29, virion(+)μNS truncated = 54, virion(+)μNS H570Q = 102, virion(+)μNS C572S = 96; SeV(−)μNS = 511, SeV(+)μNS full length = 152, SeV(+)μNS truncated = 273, SeV(+)μNS H570Q = 474, SeV(+)μNS C572S = 486; poly I:C(−)μNS = 522, poly I:C(+)μNS full length = 213, poly I:C(+)μNS truncated = 334, poly I:C(+)μNS H570Q = 498, poly I:C(+)μNS C572S = 513. Experiment was performed in triplicate; average and standard deviation are shown. *P < 0.05, **P < 0.01 (unpaired t-test).

To directly challenge our hypothesis that VFs sequester IRF3 and as such interfere with the innate immune response generated by infected cells, we capitalized on the fact that MRV VFs can be formed by expressing a single viral protein μNS^34^. A549 expressing IRF3-GFP cells were transduced to express the MRV protein μNS. 24 hours post-transduction, around 40% of cells showed μNS expression and displayed cytosolic structures reminiscent to the VFs observed during MRV infection (Fig. 8A). IRF3-GFP and μNS expressing cells were then subsequently infected with MRV virions, SeV, or transfected with poly I:C. As expected, in μNS lacking cells (cells transduced with a BacMam expressing GFP), virions and SeV infection and poly I:C treatment induced IRF3-GFP nuclear translocation (Fig. 8B), as previously observed (Fig. 6). However, upon expression of μNS and formation of VF-like structures, nuclear translocation of IRF3-GFP was significantly impaired and this occurred independently of whether the cells were infected with MRV virion, SeV or stimulated with poly I:C (Fig. 8A and B). Importantly, expression of μNS and formation of VF-like structures interfered with the immune response generated upon virion and SeV infection and poly I:C treatment, as measured by the inhibition of the transcriptional upregulation of both IFNβ1 and IFNλ2/3 (Fig. 8C). These results demonstrate that the VF protein μNS is sufficient to block IRF3 nuclear translocation which in turn interferes with IFN transcriptional activation. We then tested whether the viral factory forming capacity of μNS is required for its ability to block IRF3 nuclear translocation. There are two point mutations (570Q and C572S) within μNS which have been previously described to abolish its ability to form viral factories^35^. IRF3-GFP-expressing A549 cells were transduced with lentiviruses expressing full length μNS, a truncated μNS which is still capable of forming factories or μNS containing single point mutations that abolish VF formation (Fig. 9A). These cells were then infected with MRV virions, SeV or transfected with poly I:C. We determined that μNS’s ability to restrict IRF3 nuclear translocation correlates with its ability to form VFs. As expected (Fig. 8B), expression of μNS full length strongly impaired IRF3 nuclear translocation upon virion and SeV infection or poly I:C treatment. Similar results were obtained when expressing a N-terminal truncated version of μNS which retain the capacity of forming VFs (Fig. 9A,B). On the contrary, full-length version of μNS that loss their capacity to form VFs were not able to prevent IRF3 nuclear translocation upon virion and SeV infection or poly I:C treatment (Fig. 9B). All together, these results show that MRV can inhibit the antiviral immune induction through the μNS-dependent sequestration of the transcription factor IRF3 into its viral factories.

## Discussion

In this study, we have evaluated MRV’s strategy to combat detection and clearance by the host antiviral intrinsic innate immune response. We show that although both MRV virions and ISVPs are equally sensed by the intrinsic innate immune pathway leading to the activation of IRF3 by phosphorylation, ISVPs induce a reduced antiviral immune response compared to their virion counterparts. We determined that ISVP infection fully blocks nuclear translocation of IRF3 strongly interfering with the production of both type I (IFNβ1) and III (IFNλ2/3) interferons. We found that IRF3 was sequestered into MRV viral factories and demonstrated that the viral factory protein μNS alone was sufficient for sequestering IRF3 and blocking the antiviral immune induction. Interestingly, by determining the time necessary for MRV to mount this μNS-mediated anti-immune strategy, we found that virions also block IRF3 translocation but with a much slower kinetics and efficiency compared to ISVPs. This difference in kinetics of IRF3 sequestration in viral factories is intrinsic to the slower kinetics of virion-replication initiation and viral factory formation compared to ISVPs and explains why ISVPs induce a much reduced antiviral innate immune response compared to infection by virions. In conclusion, we have unraveled a novel strategy developed by MRV to interfere with the production of IFNs by sequestering IRF3 in viral factories. Additionally, to our knowledge, our findings clearly demonstrate for the first time, the function of viral factories as immune evasion strategy.

IRF3 is a critical member in the intrinsic immune system. Its activation is downstream of three major pathogen sensing pathways: TLR, RLR, and STING. After its phosphorylation by TBK1 it becomes activated, dimerizes, and is then transported into the nucleus to drive the production of both interferons and handful of interferon stimulated genes. This places IRF3 as a critical obstacle, which most viruses must overcome to establish a persistent infection within the host. MRV virions have been previously shown to be a stimulator of IRF3 causing its phosphorylation and nuclear localization^23^ and the production of IFN^36^. While these studies showed a greater nuclear translocation than our study, the MOI used in the previous study was much greater than ours (MOI 500 vs 1). Additionally, Holms, *et al*.^23^. demonstrated that the activation of IRF3 occurs from the sensing of incoming viruses and that synthesis of *de novo* viral RNA is not necessary for stimulation, similar to our results showing that UV inactivated particles have a higher immune detection (Fig. 2). Interestingly, they determined that infection of IRF3^−/−^ Mouse embryonic fibroblasts (MEFs) was similar to their WT counterparts at early times post infection however over a longer term infection MEFs lacking IRF3 had more viral spread and increased apoptosis^23, 32^. The increased apoptosis is due to the expression of Noxa, a pro-apoptotic member of the BCL-2 family, which is induced by IRF3 late during viral infection^37^. As such, blocking IRF3 nuclear translocation might confer an advantage for MRV as it will block both the IFN production and the pro-apoptotic response. ISVP infection results in the faster formation of viral factories (Figure S1), thereby promoting a faster and more efficient sequestration of IRF3 giving ISVPs a replication advantage compared to virions. We have previously shown that ISVP infection leads to the production of TGFβ, acting as a pro-survival cytokine; as such it appears that ISVP confers an infectious advantage compared to their virion counterparts by using complementary strategies (*i.e*. IRF3 sequestration into viral factories and production of the pro-survival factor TGFβ).

The IFN-mediated antiviral response can be divided into two signaling layers. In the first sensing loop PRRs recognize pathogen intrusion and initiate signaling which leads to the production of IFN and some ISGs. This signal transduction relies on the activation of IRF3. The second amplification loop is initiated when IFN binds to its receptor and induces a JAK/STAT signaling pathway leading to the activation of IRF9. IRF9 is a critical member of the ISGF3 complex along with STAT1/STAT2, which promote transcription of hundreds of ISGs. For MRV there have been previous reports demonstrating the ability to down regulate the antiviral innate immune induction^38^. This property has been mapped to the viral protein μ2, which has been shown to act upon IRF9 causing its nuclear accumulation and thereby reducing its ability to produce an immune response after IFN stimulation, however the mechanism by which μ2 exerts is action is unknown^27, 28^. Here we demonstrate that MRV is also able to interfere with the IFN-mediated antiviral response by blocking nuclear translocation of IRF3. The ability of MRV to block both the sensing loop and the amplification loop of IFN signaling ensures an efficient inhibition of the antiviral response. Indeed the IFN receptor and PRRs (*e.g*. RLR and the signaling adaptor MAVS) are themselves ISGs^39^. Additionally, it was reported that in some cell types production of IFN, upon MRV infection, was only partially dependent on IRF3^36^. As such blocking the downstream IRF9-dependent amplification loop still allows MRV to interefere with the antiviral response.

Blocking the IFN-mediated antiviral response by interfering with both the sensing and amplification loop of IFN signaling is a common strategy used by other members of the *Reoviridae* family. The non-structural protein NSP1 from rotaviruses has developed a method to target multiple IRF family members. IRFs are composed of a DNA binding domain and an IRF association domain (IAD). The IAD domain is the region, which allows for dimerization to occur between IRFs. To efficiently shut down the antiviral innate immune response, NSP1 binds the IAD region of IRF3, IRF5, IRF7 and IRF9 and targets these IRFs for proteasomal degradation^24, 25^. IRF proteins in the inactive stage exist as a monomer. Upon activation/phosphorylation of the key serine residues the protein unfolds exposing contact points allowing for dimer formation. Rotavirus has been shown to target both the monomeric and dimeric forms of the IRF proteins thereby allowing for downregulation prior to viral detection^25^. Additionally, rotavirus has been shown to block the nuclear import of activated STAT1 and STAT2 molecules, however it was never directly shown whether this block in nuclear accumulation was due to the loss of IRF9^40^. Like MRV, this efficient strategy ensures that both the sensing and amplification loop of IFN signaling is impaired during rotavirus infection.

Our data show that to mount an antiviral strategy MRV replication is required, which is also the case for rotavirus^24^. However, other viruses are able to block antiviral response by delivering proteins during infection which directly interfere with IFN-signaling. Varicella-zoster virus (VZV) is an alphaherpesvirus that has been shown to block IRF3 activation. VZV immediate-early protein 62 (IE62), which is packaged and present in the viral tegument, can block the phosphorylation of IRF3 by TBK1^41^. IE62 does not block the interaction between TBK1 and IRF3 and therefore acts as a “sink” to prevent further activation and interferon production.

Within the arms race between cellular antiviral strategies and viral immune evasion strategies, the faster viruses initiate countermeasures the more likely the virus can persist. We found that both MRV virions and ISVPs can interfere with IRF3-mediated signaling but with distinct efficiency and kinetics. Virions are much less efficient and slower compared to ISVPs. We have previously shown that ISVPs initiate cytosolic replication very rapidly as they are directly released from the endocytic vesicles^14^. On the contrary, virions require trafficking and processing into the early and late endosomal compartments^42^ to be activated by cellular endosomal proteases^20, 43^, thereby delaying their cytoplasmic delivery and initiation of replication. As this immune evastion strategy is replication dependent (Fig. 2), a delayed entry kinetics results in a delayed sequestration of IRF3. The difference in efficiency between virion and ISVPs is explained by virions and ISVPs displaying a different specific infectivity (particle:pfu ratio^22^). The role of ISVPs has often been overlooked as they only initiate the first round of infection, and all further rounds of infection are initiated by virions. We have shown here, as well as in our previous work, that ISVPs are not only beneficial by blocking the immune induction allowing for a greater infection but they also secrete pro-survival compounds to allowing for cell survival and increased virus production.

All cytosolic replicating RNA viruses create viral induced replication compartments or viral factories. Viral factories are important to concentrate viral components avoiding dilution within the cytosol and as such promoting progeny virus assembly. It has been proposed that they also act as a shield to protect viral genomes from the recognition by the intrinsic antiviral immune system. However, although this model is very attractive there is little to no evidence of this activity. The structural backbone of the MRV viral factories is made up of the non-structural protein μNS. μNS is a 80 kDa protein which is necessary and sufficient for the formation of viral factories (Figs 8, 9, and ref. 34). μNS is binds the capsid components (λ1, λ2, λ3, μ2, and σ2) and the non-structural protein σNS^44, 45^ and as such is the core coordinator of virus replication and assembly (Fig. 9A). Additionally, μNS has been shown to bind to both clathrin heavy and light chains thereby reducing endocytosis within infected cells^33^, which in turn blocks superinfection. Here by overexpressing μNS alone which results in the formation of virus factory-like structures we show that μNS is also able to sequester IRF3, blocking its nuclear translocation and as such blocking IFN-mediated response (Figs 7 and 8). Additionally, we show that the viral factory forming capacity of μNS is required for this sequestration (Fig. 9). This clearly demonstrates, to the best of our knowledge, for the first time that virus factories act as a point of immune evasion. This places μNS as a critical player in the MRV lifecycle, it on the one hand provides a scaffolding to co-ordinate viral replication and assembly and on the other hand provides a safety net which protects the virus against host interference (preventing superinfection by blocking clathrin and blocking immune induction by binding IRF3).

In conclusion, this work describes a novel strategy by MRV to interfere with the intrinsic antiviral immune system and clearly demonstrates that viral factories act as an immune evasion strategy as such placing viral factories as a potential target for antiviral intervention.

## Materials and Methods

### Cell and Viruses

A549 cells (ATCC) were maintained in DMEM with 10% fetal bovine serum, 1% penicillin/streptomycin and 0.5 mM HEPES. A549 cells expressing IRF3-GFP or GFP were produced by puromycin selection following transduction of A549 cells with a lentivirus vector (pWPI) expressing IRF3-GFP or GFP. Individual single cell clones were selected, grown and confirmed to act similar to WT cells. 293 T HEK cells (ATCC) were maintained in IMDM with 10% fetal bovine serum, 1% penicillin/streptomycin. Reovirus strains Type 3 clone 9 (T3C9) and T1L derived from stocks originally obtained from Bernard N. Fields were grown and purified by standard protocols^22, 46^. ISVPs were generated by diluting purified virion particles 1:20 in virion buffer (150 mM NaCl, 10 mM MgCl2, 10 mM Tris, pH 7.5) in the presence of 200 µg/mL chymotrypsin (Sigma-Aldrich) at 32 °C for 15 min. The reaction was stopped by addition to serum containing media or addition of 1 mM PMSF (Sigma-Aldrich). Sendai virus (Cantell strain) was a kind gift from Marco Binder. The infectivity of virions and ISVPs was quantified in A549 cells using an In-cell western approach^22, 47^. All MRV infections were performed at an MOI of 1 or 3 as indicated in figure legend and Sendai virus was used at an MOI of 3. For MRV co-infection experiments each virus was added at an MOI of 1. UV inactivation was performed by diluting MRV T3C9 virions or ISVPs 1:10 in medium and exposing them to 6,000 J of UV irradiation prior to infection. Viral infections were performed on cells seeded 24 hours prior to infection. Media was removed from cells and new media containing viruses was added. Viruses were maintained during the entire course of infection. Samples (RNA/protein/IF) were harvested at times indicated in figure legends.

The Gateway cloning system (Invitrogen) was used to shuffle the µNS or GFP gene into a gateway compatible baculovirus expression system according to manufacturer instructions (Invitrogen). The µNS-BacMam or GFP-BacMam was delivered into cells using the Virapower BacMam expression system (Life Technologies). Bacmids encoding wt-µNS or GFP were engineered using the Gateway system and following the manufacturer’s instructions (Life Technologies). Baculoviruses, were obtained by transfecting Sf9 cells with the bacmids and passaging the virus three times in Sf9 cells to sufficiently high titres. Baculoviruses obtained from the third passage were used to transduce A549 cells.

The Gateway cloning system (Invitrogen) was used to shuffle RFP, the full length µNS or truncated µNS into a gateway compatible lentivirus (pWPI) expression system according to manufacturer instructions (Invitrogen). The two point mutations H570Q and C572S were created through site-directed mutagenesis of the Gateway pEntry clone according to manufacturer instructions (Qiagen) using the following primers (H570Q, for gtatttgagacaccaGacctgcattaatggtcatac; rev, gtatgaccattaatgcaggtCtggtgtctcaaatac) and (C572S, for gtatttgagacaccacaccAgcattaatggtcatac; rev, gtatgaccattaatgcTggtgtggtgtctcaaatac). Lentiviruses were produced by PEI transfection of HEK cells. Supernatants containing lentiviruses were harvested 72 hours post-transfection. A549 IRF3-GFP cells were seeded into 24-well plates and were transduced with supernatant containing lentiviruses. 72 hours post-transduction cells were infected or transfected as described above.

### Antibodies/Reagents

Rabbit polyclonal antibody against reovirus μNS was used at 1:1000 for immunostaining and Western blots^22^. Commercially available primary antibodies were mouse polyclonal antibody against β-actin (Santa Cruz Biotechnology), rabbit polyclonal antibody against MAVS (Enzo), rabbit polyclonal antibody against phosho-IRF3 (Cell Signaling), rabbit polyclonal antibody against IRF3 (Santa Cruz Biotechnology). Secondary antibodies were conjugated with AF568 (Molecular Probes) directed against the animal source. Anti-mouse (GE Healthcare # NA934V) and anti-rabbit (GE Healthcare #NA931V) antibodies, each coupled with horseradish peroxidase were used as secondary antibodies for Western blot at a 1:5000 dilution. HMW and LMW poly I:C (Peprotech) were used in a 50:50 ratio at a final concentration of 1 µg/mL and were transfected using Lipofectamine according to manufacturer’s instructions.

### RNA isolation, cDNA, and qPCR

RNA was harvested from cells using NuceloSpin RNA extraction kit (Macherey-Nagel) as per manufacturer’s instructions. cDNA was made using iScript reverse transcriptase (BioRad) from 250 ng of total RNA as per manufacturer’s instructions. q-RT-PCR was performed using SsoAdvanced SYBR green (BioRad) as per manufacturer’s instructions, TBP and HPRT1 were used as normalizing genes^22^.

### Western blot

At time of harvest, cell media was removed, cells were rinsed once with 1X PBS and lysed with 1X RIPA (150 mM sodium chloride, 1.0% Triton X-100, 0.5% sodium deoxycholate, 0.1% sodium dodecyl sulphate (SDS), 50 mM Tris, pH 8.0 with phosphatase and protease inhibitors (Sigma-Aldrich)) for 5 mins at room temperature (RT). Lysates were collected and equal protein amounts were run on 12% SDS-PAGE at 120 V for 1.5–2 h. Proteins were transferred to nitrocellulose membranes at 100 V for 1 h in the cold. Membranes were blocked overnight in 5% milk/PBS at 4°C. Primary antibodies were diluted in 5% milk/PBS and were incubated at RT for 2 h with rocking. Membranes were washed 3X in PBS + 0.05% Tween-20 (PBST) for 5 mins at RT. Secondary antibodies were diluted in 5% milk/PBS and incubated at RT for 1 h with rocking. Membranes were washed 3X in PBST for 5 mins at RT. HRP detection reagent (GE Healthcare) was mixed 1:1 and incubated at RT for 5 mins. Membranes were exposed to film and developed.

### Indirect immunofluorescence assay

Cells grown on glass coverslips were fixed in 2% paraformaldehyde (PFA) for 20 mins at RT. Cells were washed with phosphate-buffered saline (PBS) and permeabilized in PBS containing 0.5% Triton-X for 15 mins at RT. Primary antibodies were diluted in 1% BSA/PBS and incubated for 1 h at RT. Coverslips were washed in 1X PBS three times and incubated with secondary antibodies diluted in 1% BSA/PBS for 45 mins at RT. Coverslips were washed in 1X PBS three times and mounted on glass slides with ProLong Gold containing DAPI (Molecular Probes). Cells were imaged by epifluorescence on a Nikon Eclipse Ti-S (Nikon).

## Electronic supplementary material

Supplementary Information
