# Supplementary material for: Reovirus inhibits interferon production by sequestering IRF3 into viral factories
Source: Sci Rep. 2017 Sep 7;7:10873. doi: 10.1038/s41598-017-11469-6 (PMC5589761; doi:10.1038/s41598-017-11469-6)

Reovirus inhibits interferon production by sequestering IRF3 into viral factories

Megan L. Stanifer[1], Christian Kischnick[1], Anja Rippert[1], Dorothee Albrecht[2], Steeve Boulant[1,2*]

[1] Department of Infectious Diseases, Virology, Heidelberg University, Germany; [2] Schaller research group at CellNetworks and German Cancer Research Center (DKFZ), Research Group "Cellular polarity and viral infection" (F140), Heidelberg, Germany

Running Title: Reovirus interferes with intrinsic innate immune response

[*]Corresponding author

Steeve Boulant, Ph.D.

Department of Infectious Disease, Virology

Schaller research group at CellNetworks and DKFZ

Heidelberg University

Im Neuenheimer Feld 581

69120 Heidelberg, Germany

Phone: +49 (0) 6221 42 1560

Fax: +49 (0) 6221 42 1560

Email: s.boulant@dkfz.de

Supplementary figure legends

Figure S1. ISVP infection initiates faster compared to virion infection. A549 cells were infected with reovirus MRV virions or ISVPs at MOI=1. Virus infection was monitored at indicated time pointes by (A.) Fluorescence microscopy following immunostaining of the non-structural viral protein μNS (red). Cell nuclei were stained using DAPI (blue). Experiment was performed in triplicate; representative images are shown. (B). Western blot using an anti-μNS antibody. Actin is used as a loading control. Experiment was performed in triplicate; representative images are shown**.**

Figure S2. μNS sequesters IRF3 into VFs. (A) GFP expressing A549 cells were infected with MRV virions and ISVPs both at an MOI=3. 16 hours post-infection, immunostaining of the non-structural viral protein μNS (red) was used to monitor the appearance of viral factories. Cell nuclei were stained using DAPI (blue). GFP expression remains uniform and is not sequestered into the viral factories on its own. Experiment was performed in triplicate; representative images are shown. (B). A549 cells were infected with MRV virions and ISVPs both at an MOI=3. 16 hours post-infection, fluorescent microscopy was used to monitor the localization of endogenous IRF3 using an anti-IRF3 antibody (red). Cell nuclei were stained using DAPI (blue). (Left panel) To ensure that the signal detected for IRF3 was not a crosstalk or cross-reactivity with the anti-μNS antibody, cells were immunostained only with the anti-IRF3 antibody. (Right panel) To confirm that all cells were infected in images in left panel, 16 hours post-infection, cells were immunostained with an anti-μNS antibody (red) and cell nuclei were stained using DAPI (blue). Experiment was performed in triplicate; representative images are shown**.**

Figure S3. IRF3 sequestration into MRV VFs is strain independent. IRF3-GFP expressing A549 cells were infected with T1L MRV virions or ISVPs at MOI=3. Virus

infection was monitored 16 hours post-infection by fluorescence microscopy following immunostaining of the non-structural viral protein μNS (red). Cell nuclei were stained using DAPI (blue). Experiment was performed in triplicate; representative images are shown.

Supplementary Figure 1

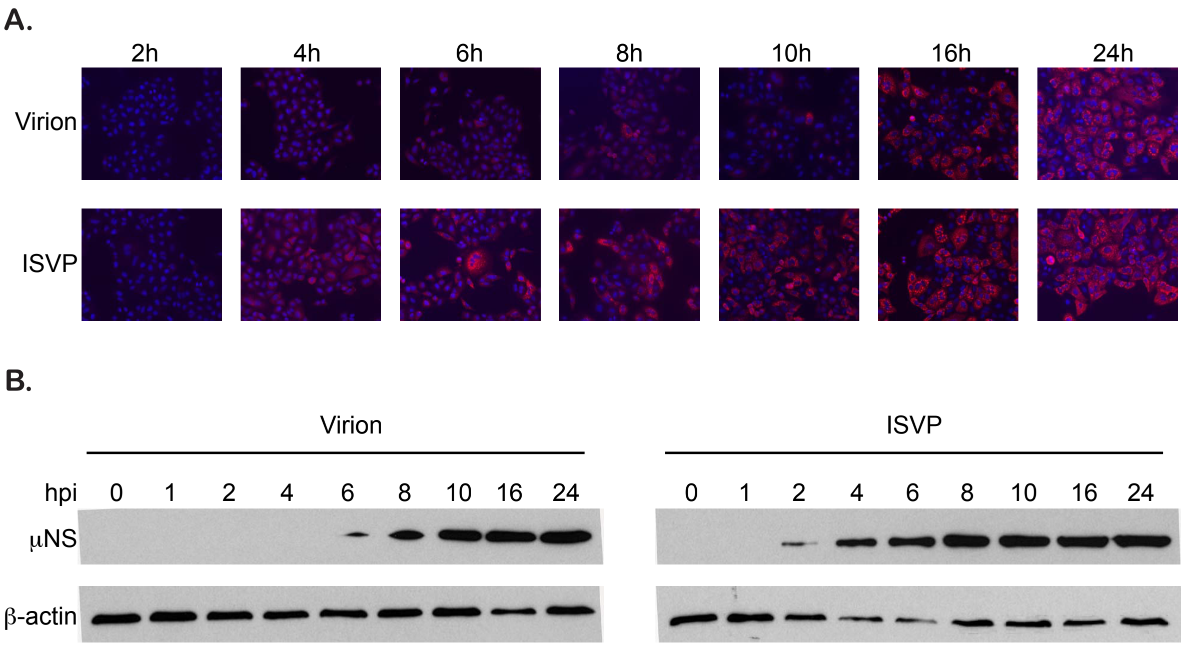

Supplementary Figure 2

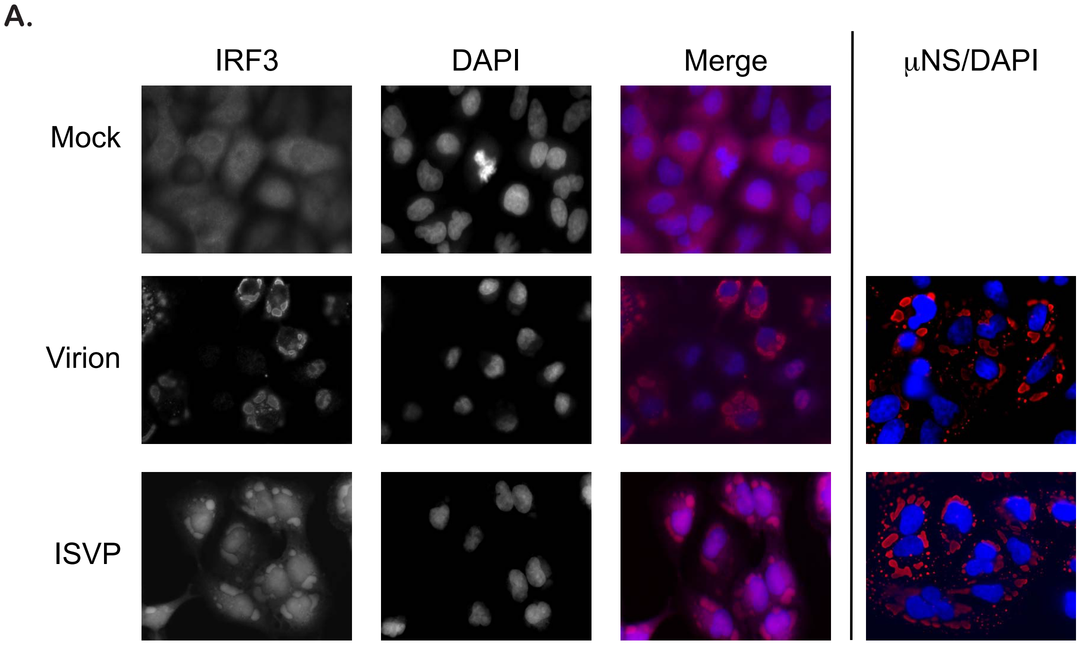

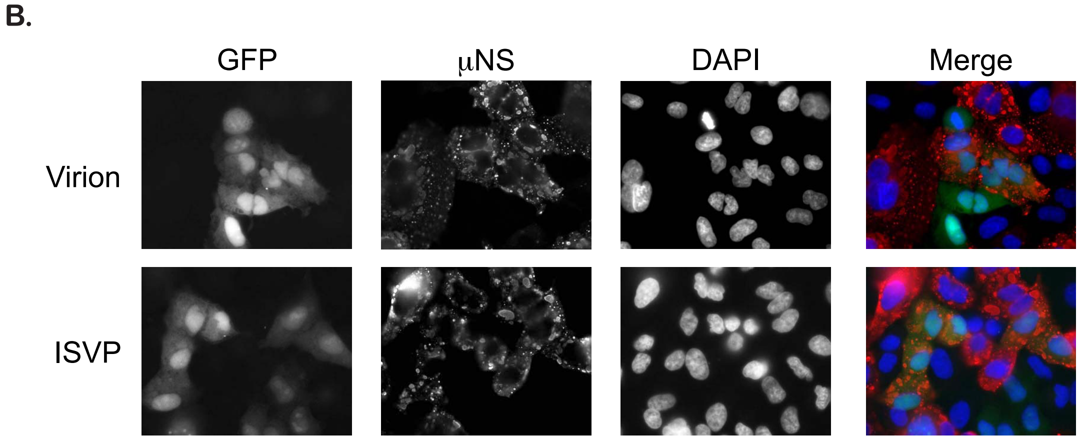

Supplementary Figure 3

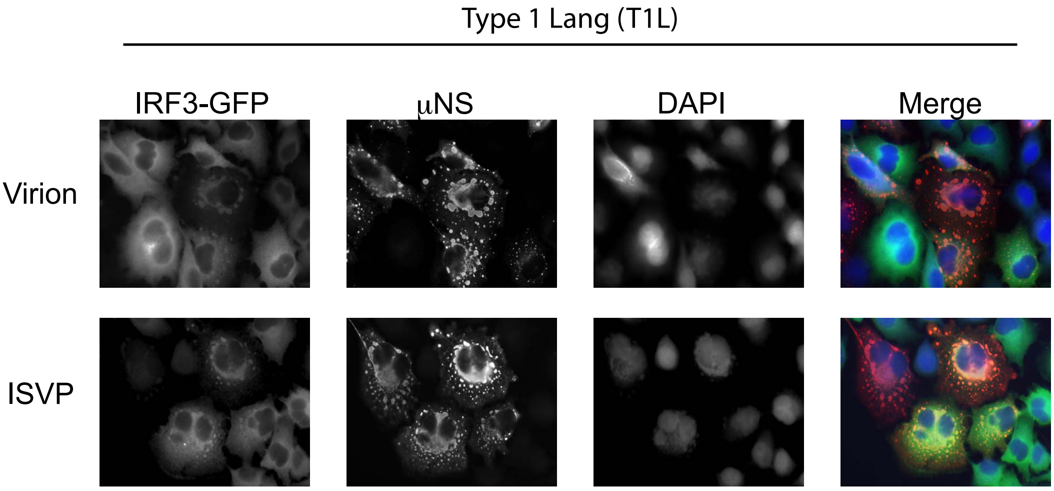

Full scans of blots from Figure 4

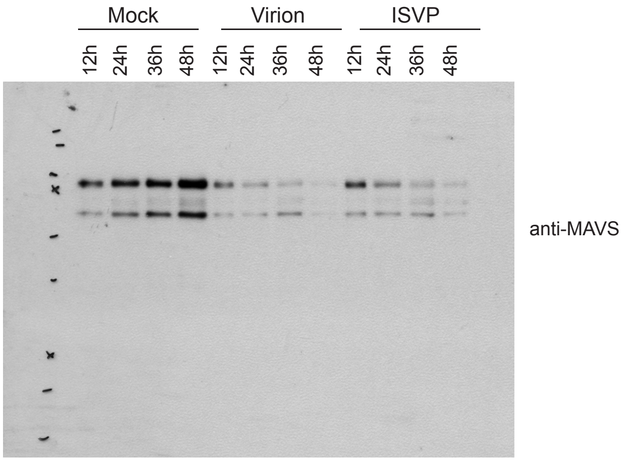

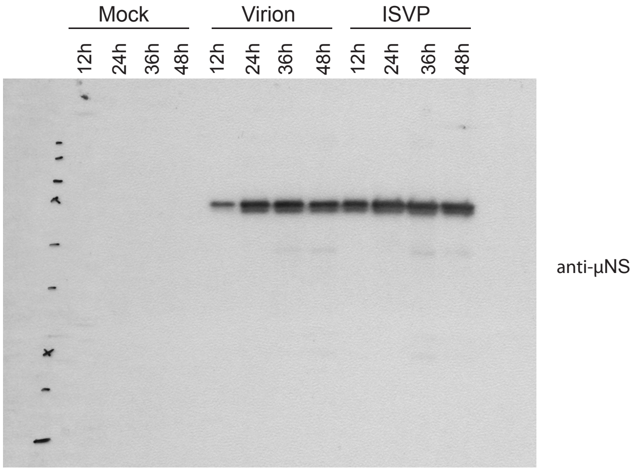

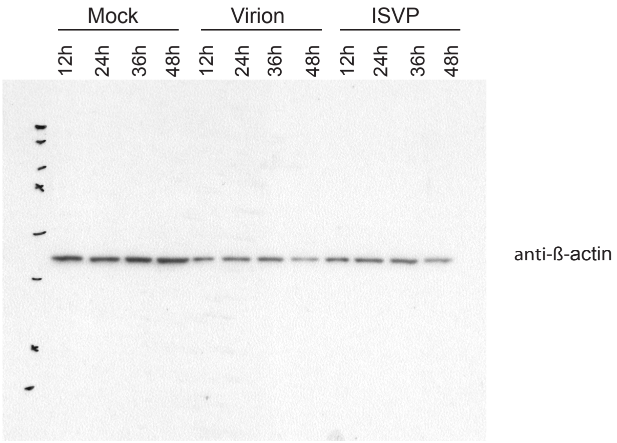

Full scans of blots from Figure 5

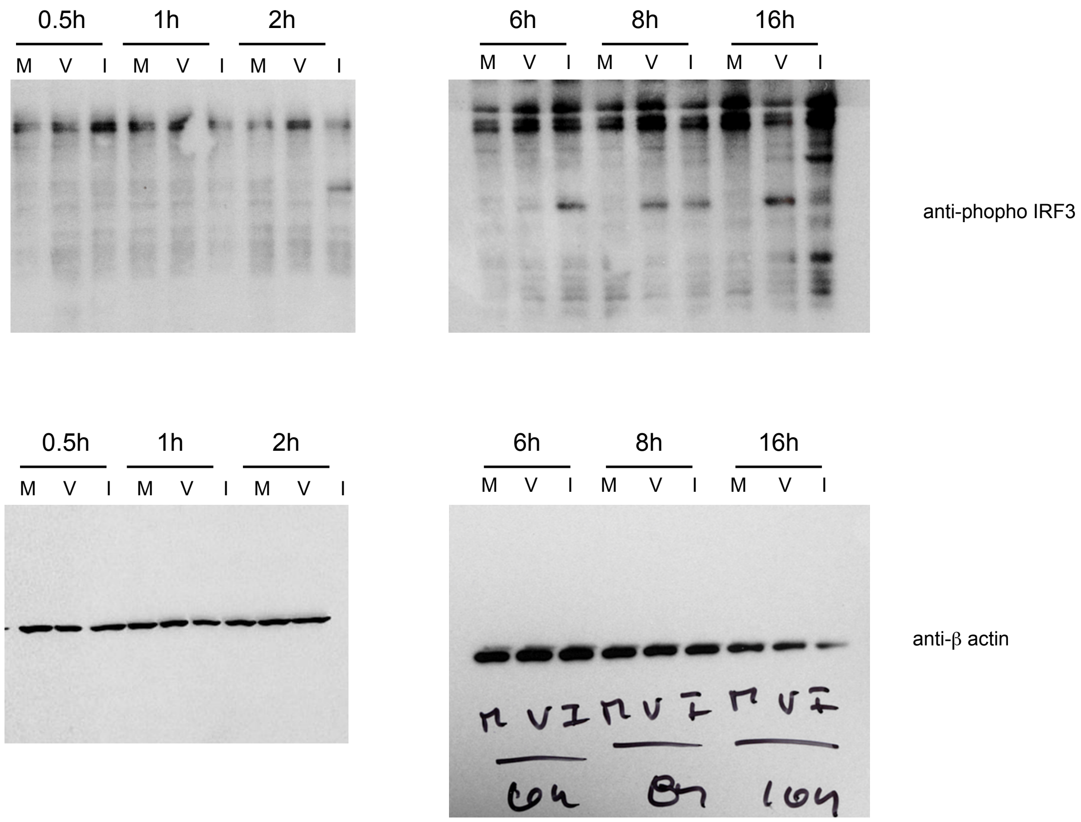

Supplement: Supplementary file 1 — Supplementary Information [file 41598_2017_11469_MOESM1_ESM.pdf]
